# TRIM11, a direct target of miR-24-3p, promotes cell proliferation and inhibits apoptosis in colon cancer

**DOI:** 10.18632/oncotarget.13550

**Published:** 2016-11-24

**Authors:** Yan Yin, Jun Zhong, Si-Wei Li, Jian-Zhe Li, Min Zhou, Yin Chen, Yi Sang, Lijuan Liu

**Affiliations:** ^1^ Department of Pharmacy, Jiangxi Cancer Hospital, Nanchang, China; ^2^ Department of Radiotherapy, Jiangxi Cancer Hospital, Nanchang, China; ^3^ Department of Radiation Oncology, The Affiliated Hospital of Guilin Medical University, Guilin, China; ^4^ Department of Pharmacy, Ruikang Hospital, Guangxi University of Chinese Medicine, Nanning, China; ^5^ Nanchang Key Laboratory of Cancer Pathogenesis and Translational Research, Center Laboratory, The Third Affiliated Hospital, Nanchang University, Nanchang, China

**Keywords:** TRIM11, colon cancer, miR-24-3p

## Abstract

TRIM11 (tripartite motif-containing protein 11) is an E3 ubiquitin ligase recently identified as an oncogene in malignant glioma and lung cancer. In the present study, we report that expression of TRIM11 was increased in colon cancer (CC) tissue relative to paired normal tissues and that higher TRIM11 levels predicted poor overall survival (OS) and disease-free survival (DFS) in CC patients. Mechanistically, we showed that miR-24-3p downregulation contributes to TRIM11 upregulation in CC. We also demonstrated that TRIM11 overexpression promotes cell proliferation and colony formation and inhibits apoptosis in CC, while knocking down TRIM11 using CRISPR/Cas9-mediated genome editing inhibited cell proliferation and induced apoptosis. Silencing TRIM11 *in vivo* decreased tumor growth. These findings indicate that TRIM11 facilitates CC progression by promoting cell proliferation and inhibiting apoptosis and that the novel miR-24-3p/TRIM11 axis may be a useful new target for treating patients with CC.

## INTRODUCTION

Colon cancer (CC) is the third most common cancer and the fourth leading cause of cancer-related deaths worldwide [1–3]. Mortality remains high despite improvements in prevention and treatment. CC, therefore, remains a major health problem. The dysregulation of oncogenes or tumor suppressor genes is tightly correlated with CC initiation, progression, and resistance to therapy, of all of which involve changes in the biological characteristics of cancer cells, including cell growth, apoptosis, migration, invasion, and metabolism [4, 5]. The identification of novel therapeutic targets, less toxic therapies, and better predictive markers is urgently needed.

TRIMs, members of the RING family of Ub E3 ligases, are characterized by the presence of three conserved domains, RING, B-Box, and coiled-coil (RBCC) [6]. TRIM family proteins are involved in many biological processes, and changes in their abundance or activity are associated with several pathological conditions, including viral infections, developmental and neurodegenerative disorders, and cancers [7, 8]. TRIM11 binds to and destabilizes Humanin, an inhibitor of Alzheimer-like neuronal insults [9]. TRIM11 destabilization of the activator-mediated cofactor complex (ARC105) suppresses ARC105-mediated transcriptional activation induced by transforming growth factor β signaling [10]. Trim11 promotes Pax6 degradation, and the subsequent inhibition of Pax6 transcriptional activity impairs neurogenesis [11]. TRIM11 interacts with Phox2b, a homeodomain transcription factor that modulates the development of noradrenergic neurons, and increases expression of dopamine β-hydroxylase gene [12]. In addition, TRIM11 is upregulated in malignant gliomas, where it promotes proliferation, invasion, migration, and tumor growth by increasing the accumulation of EGFR and activity of MAPK cascade [13]. TRIM11 is also highly expressed in lung cancer tissues and cell lines, and higher expression of TRIM11 is correlated with the poorer prognosis in lung cancer patients [14].

MicroRNAs (miRNAs) are non-coding, single-stranded RNA molecules (∼22 nucleotides in length), which post-transcriptionally regulate gene expression by binding to the 3’-untranslated region (3’-UTR) of specific mRNAs and targeting them for degradation or translational repression [15–17]. Among these microRNAs, miR-24-3p has been shown to act as a cell type specific oncogene or tumor suppressor in a variety of cancers [18–20]. Gao et al. [18] demonstrated that miR-24-3p was downregulated in human CC tissues relative to corresponding non-cancerous tissues, and overexpression of miR-24-3p suppressed CC cell proliferation, migration, and invasion *in vitro*. Mishra et al. [21] demonstrated that miR-24 functions as a tumor suppressor independent of p53 by targeting and repressing dihydrofolate reductase in CC cell lines. In addition, Fang et al. [22] showed that the plasma levels of miR-24 were decreased in patients with CC and benign lesions (polyps and adenoma) compared with healthy controls, revealing miR-24as a promising potential biomarker for CC detection. However, little is known about the targets of miR-24-3p in CC.

In this study, we evaluated the expression of TRIM11 in clinical CC tissues compared and adjacent non-cancerous tissues, and examined the relationship between TRIM11 expression and clinical outcomes. We also sought to evaluate the effect of TRIM11 expression on CC cell phenotypes *in vitro* and *in vivo*. Moreover, we examined miRNAs targeting TRIM11 in order to shed light on the mechanisms of TRIM11 regulation and dysregulation in CC.

## RESULTS

### TRIM11 expression is up-regulated in CC cells

**Figure 1:**
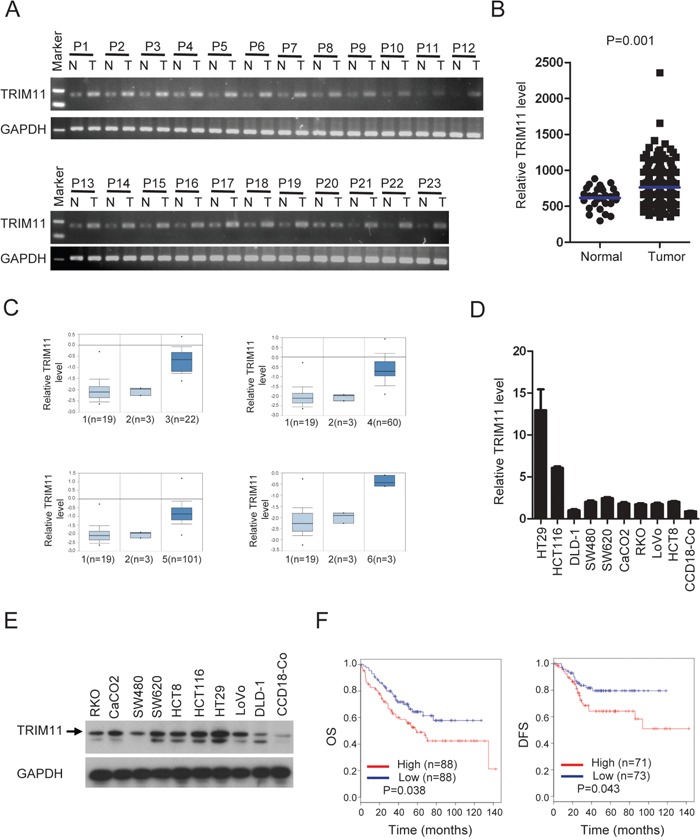
TRIM11 expression is increased in CC, and higher *TRIM11* levelspredict a poor outcome **A**. qPCR analysis of TRIM11 expression in clinical CC samples of both tumor and the paired normal tissues. **B**. Meta-analysis of *TRIM11* mRNA levels in CC samples from the MethHC database (http://methhc.mbc.nctu.edu.tw/php/index.php). Blue bars indicate mean value. The P value was calculated from the raw data using Student's t-test (P=0.001). **C**. Meta-analysis of *TRIM11* mRNA levels in CC samples from the Oncomine database (http://www.oncomine.org). Box plots showing the increased expression of *TRIM11* during tumorigenesis in CC datasets. 1: normal colon tissues, 2: normal rectum tissues, 3: cecum adenocarcinoma tissues, 4: rectal adenocarcinoma tissues, 5: colonadenocarcinoma tissues, 6: rectosigmoid adenocarcinoma tissues. The y-axis represents TRIM11 expression. Shaded boxes represent the interquartile range (25^th^–75^th^ percentile). Whiskers represent the 10^th^–90^th^ percentile. The bars denote the median. **D**. qRT-PCR analysis of TRIM11 mRNA levels cell lines. **E**. Western blot analysis of TRIM11 protein levels cell lines. **F**. CC patients with highTRIM11 expression exhibited significantly shorter overall survival OS and DFS compared with those with low expression, P <0.05.

We measured the mRNA level of TRIM11 in CC tissues and normal colon tissues using quantitative PCR (qPCR). As shown in Figure 1A and Supplementary Figure 1, the TRIM11 mRNA level was significantly higher in 22 out of 23 tumor tissues than in the paired normal colon tissues. We analyzed its expression using online databases [23], as shown in Figure 1B and 1C, and found TRIM11 is significantly up-regulated in CC tissues compared with the normal tissues (P<0.01). Likewise, we found that TRIM11 mRNA and protein levels were elevated in all of the ten CC cell lines examined, relative tothe normal colon fibroblast cell line (CCD18-Co) (Figure 1D and 1E), suggesting that TRIM11 is up-regulated in CC and may be related to CC progression.

To investigate whether TRIM11 expression can serve as a novel prognostic marker for CC patients, based on the TRIM11 expression levels reported in a large public clinical microarray database, CC samples were subdivided into two groups and the associated overall survival (OS) and disease-free survival (DFS) were analyzed. Individuals with high TRIM11 levels exhibited shorter OS and DFS than those with low levels (Figure 1F). Collectively, these results indicate that TRIM11 is up-regulated in CC and that its high expression predicts a poor outcome for CC patients.

### Mir-24-3p down-regulation is responsible for TRIM11upregulation in CC cells

**Figure 2:**
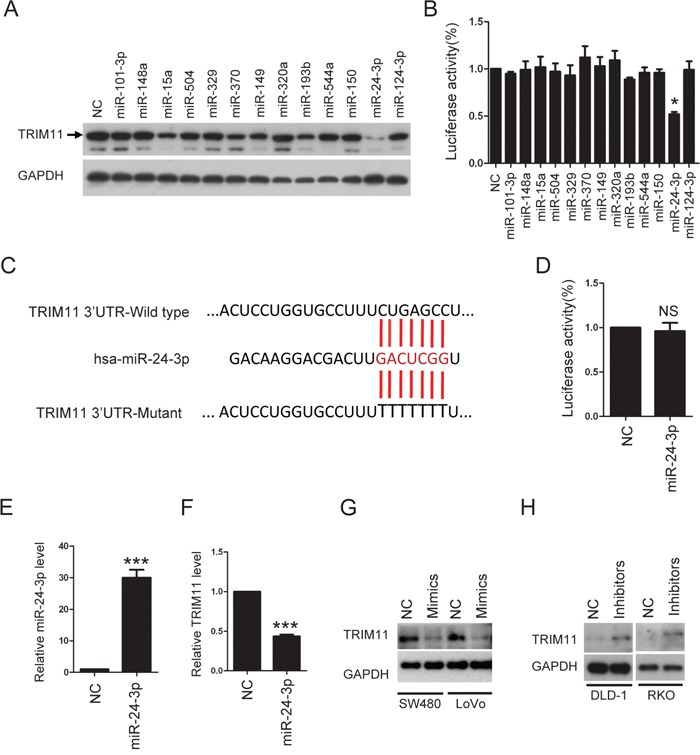
TRIM11 is direct target of miR-24-3p **A**. Western blot analysis of TRIM11 protein levels after transfection of miRNAs mimics in HCT116 cells. **B**. Luciferase activity was measured 24 h after transfection of miRNAs mimics in 293T cells. Renilla luciferase was used for normalization. The bars correspond to the mean± standard error, and the p-value was calculated using Student's t-test. *P<0.05. **C**. The sequence of miR-24-3p and the 7-mer binding site in 3’ UTR of TRIM11 mRNA. Red letters are the mutated nucleotides in the seed sequence of 3’UTR. **D**. Mutant luciferase activity was measured 24 h after transfection of miR-24-3p mimics in 293T cells. **E, F**. The levels of miR-24-3p and TRIM11 were detected after transfection of miR24-3p mimics in HCT116 cells. **G**. Western blot analysis of TRIM11 protein levels after transfection of miR24-3p mimics. **H**. Western blot analysis of TRIM11 protein levels after transfection of miR24-3p inhibitors.

To investigate how TRIM11 is up-regulated in CC cells, we first predicted which miRNAs regulated TRIM11 expression using TargetScan 5.1 (http://www.targetscan.org). Next, we selected 13 miRNAs with conserved binding to the 3’UTR of TRIM11 mRNA in multiple species. These miRNAs were transfected into HCT116 cells, and endogenous TRIM11 protein was measured by Western blotting (Figure 2A). Meanwhile, these miRNAs were co-transfected with a reporter plasmid into HCT116 cells. pGL3-luc, which contains 13 miRNAs binding sites downstream of the luciferase gene, allows for quantitative measurement of TRIM11 3’UTR activity. Figure 2A and 2B shows that miR-24-3p is the only miRNA that gave clear positive results in the two tests, indicating that miR-24-3p negatively regulates TRIM11 expression in CC cells. Importantly, mutation of the miR-24-3p seed region within the TRIM11 3’UTR abrogated the repressive ability of miR-24-3p (Figure 2C and 2D), demonstrating the specificity of the target sequence for TRIM11. Moreover, ectopic expression of miR-24-3p mimics can decrease TRIM11 mRNA level (Figure 2E and 2F). We asked whether this regulation extended to other CC cells; ectopic expression of miR-24-3p mimics also suppressed TRIM11 expression in SW480 and LoVo cells (Figure 2G). In contrast, TRIM11 protein levels increased after transfecting miR-24-3p inhibitors into DLD-1 and RKO cells (Figure 2H). These results indicate that miR-24-3p reduced the expression of TRIM11 through a direct seed sequence interaction.

### TRIM11 is inversely correlated with miR-24-3p in CC

**Figure 3:**
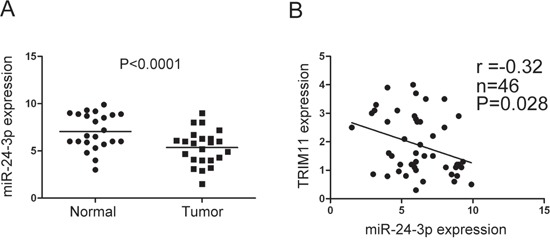
TRIM11 was negatively correlated with miR-24-3p in CC **A**. The miR-24-3p level was detected by qRT-PCR in 23 pairs of CC and corresponding non-tumor colon tissues. **B**. Pearson's correlation between miR-24-3p and TRIM11 expression levels was analyzed, showing a significant negative correlation (r = -0.32, P = 0.028).

To further verify the relationship between TRIM11 and miR-24-3p, we detected miR-24-3p expression level by qRT-PCR in the 23 pairs of CC and non-tumor colon tissues, in which TRIM11 expression level had been measured. miR-24-3p was downregulated in tumor tissues compared with normal colon tissues (Figure 3A) and TRIM11 was negatively correlated with miR-24-3p expression level (Pearson r = -0.32, P = 0.028) (Figure 3B), which suggested that the upregulated TRIM11 was, at least in part, due to downregulated miR-24-3p in CC.

### Overexpression of TRIM11 promotes CC cell proliferation and inhibits apoptosis

**Figure 4:**
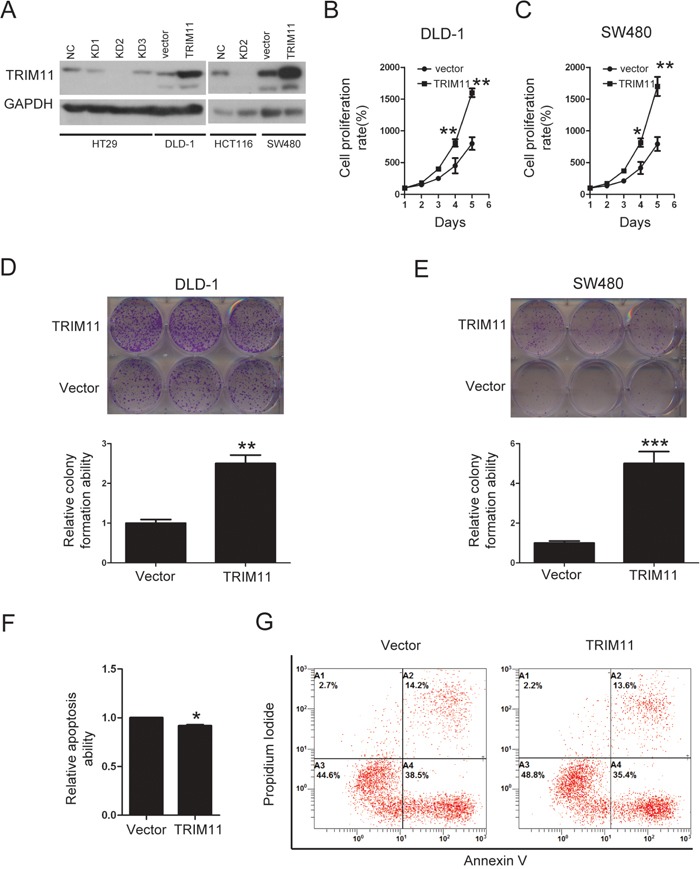
Overexpression of TRIM11 promotes CC cell proliferation and inhibits cell apoptosis **A**. The generation of stable cell lines in which TRIM11 was overexpressed or silenced was confirmed by western blotting. GAPDH was used as the internal control. **B, C**. The cell proliferation of the indicated stable cell lines *in vitro* was measured at different time points, as indicated by the CCK-8 assay. The bars correspond to the mean± standard error, and the P value was calculated using Student's t-test. *P<0.05, **P<0.01. **D, E**. The colony formation of the indicated stable cell lines *in vitro* was measured for 14 days. The bars correspond to the mean± standard error, and the p-value was calculated using Student's t-test. **P<0.01,***P<0.001. **F, G**. The stable cell lines overexpressing the empty vector or TRIM11 were treated with 150 μg/ml5-FU for 24 h and then subjected to annexin V-FITC and PI staining. Cell apoptosis was evaluated through FACS. The bars correspond to the mean± standard error (n = 3), and the P-value was calculated using Student's t-test. *P<0.05.

We stably overexpressed TRIM11 in CC cell lines (Figure 4A). During cell culture, we noticed that TRIM11-overexpressing cells proliferated faster than control counterparts. Accordingly, a significant increase in the proliferation of TRIM11 overexpressing cells compared to control cells was observed by cell counting kit-8 (CCK-8) assay (Figure 4B and 4C). Colony formation assay also indicated that overexpression of TRIM11 significantly promoted cell colony formation ability (Figure 4D and 4E).

Induction of apoptosis is a therapeutic strategy for CC treatment. To address whether TRIM11 regulated CC cell apoptosis, the propidium iodide-annexin V assay was performed; after treating DLD-1 cells with 5-FU for 24 hours, 15% of control cells underwent apoptosis, while the rate among TRIM11-expressing cells was 10% (Figure 4F and 4G). Taken together, these findings indicated that overexpression of TRIM11 promotes cell proliferation and inhibits apoptosis in CC.

### Silencing TRIM11 suppresses CC cell proliferation and induces apoptosis

**Figure 5:**
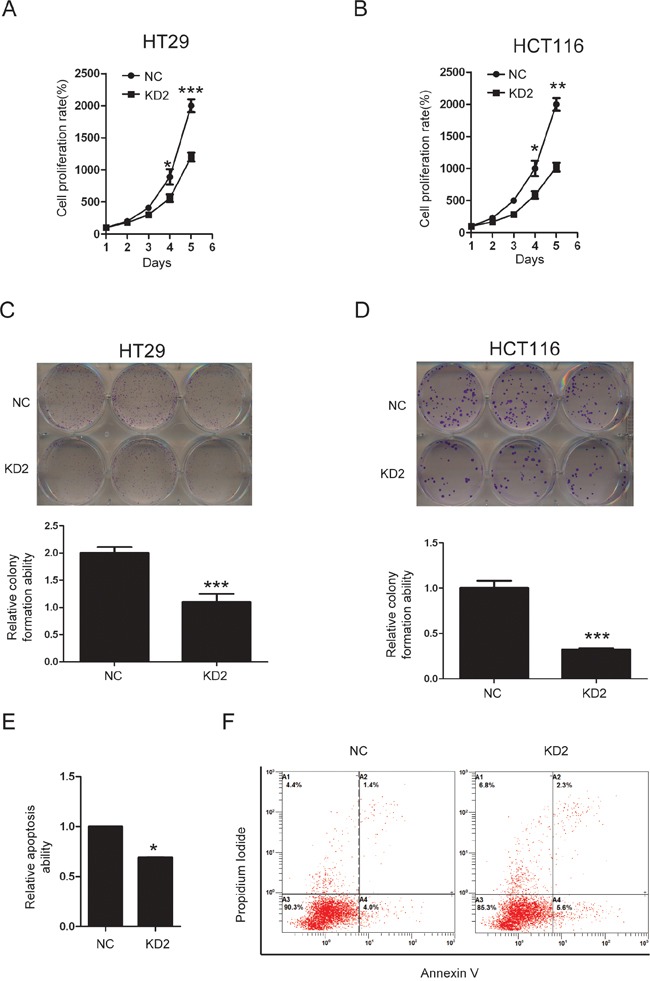
Knockdown of TRIM11 suppressed CC cell proliferation and induced apoptosis **A, B**. The cell proliferation of the indicated stable cell lines *in vitro* was measured at different time points, as indicated by the CCK-8 assay. The bars correspond to the mean± standard error, and the P value was calculated using Student's t-test. *P<0.05, **P<0.01. **C, D**. The colony formation of the indicated stable cell lines *in vitro* was measured for 14 days, as described in the Methods. The bars correspond to the mean± standard error, and the p-value was calculated using Student's t-test. **P<0.01,***P<0.001. **E, F**. The stable cell lines silencing the negative control or TRIM11 were treated with 150 μg/ml5-FU for 24 h and then subjected to annexin V-FITC and PI staining. Cell apoptosis was evaluated through FACS. The bars correspond to the mean± standard error (n = 3), and the p-value was calculated using Student's t-test. *P<0.05.

The ability to knock out genes by CRISPR/Cas9 mediated genome editing is revolutionizing modern genetics [24]. We constructed three sgRNAs targeting different regions in one of the first few exons of the human TRIM11 gene, using bioinformatics prediction to avoid obvious potential off-target effects [25]. Western blotting showed that only sgRNA#2(KD2) efficiently knocked down TRIM11 in HT29 and HCT116 cells (Figure 4A). During cell culture, we noticed that KD2 cells proliferated more slowly than the control counterpart. Furthermore, CCK-8 assay indicated that silencing TRIM11 significantly suppressed cell proliferation (Figure 5A and 5B). The colony formation assay showed that silencing TRIM11 decreased colony formation ability (Figure 5C and 5D). Furthermore, the propidium iodide-annexin V assay revealed that knockdown of TRIM11 increased the apoptosis rate after treating with 5-FU for 24 hours (Figure 5E and 5F). Collectively, our result suggests that reduced TRIM11 suppresses CC cell proliferation and induces apoptosis *in vitro*.

### Silencing TRIM11 decreased tumor growth *in vivo*

**Figure 6:**
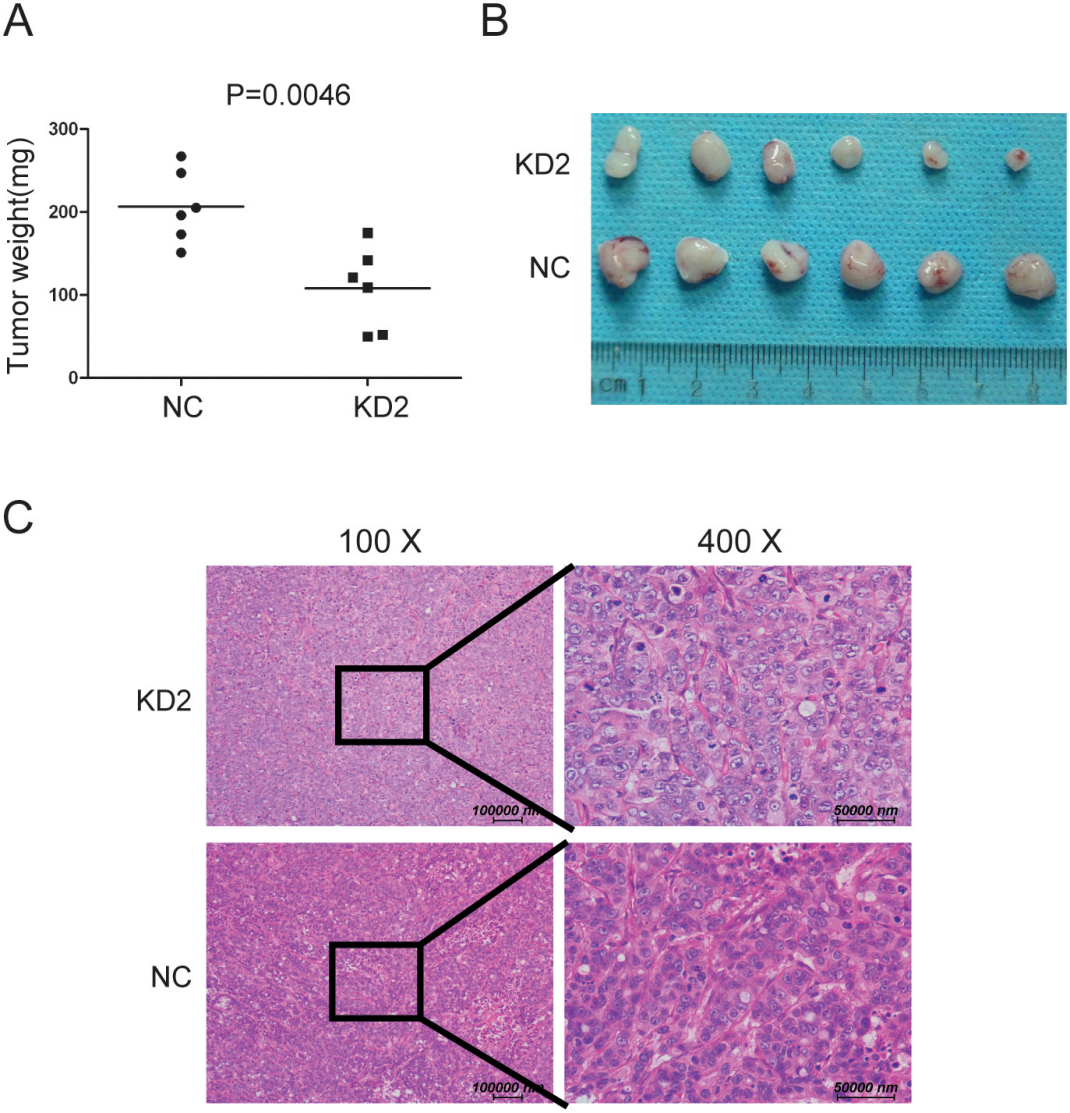
Silencing TRIM11 reduces tumor growth *in vivo* **A**. Tumor weights for the tumors formed by the indicated cells (p=0.0046). **B**. Representative images of the tumors from all the mice in each group. **C**. H&E staining representative images of tumor formed by KD2 and NC cells.

To test whether knocking down TRIM11 suppressed CC tumor growth *in vivo*, we inoculated nude mice with HT29 and its derived cells. After 4 weeks, the tumors formed by the control cells were larger and heavier than the TRIM11-silenced tumors, (Figure 6A-6C). Together, these findings indicated that silencing TRIM11 decreased tumor growth *in vivo*.

## DISCUSSION

A better understanding of the mechanisms underlying CC development, progression, and therapy resistance is urgently needed to guide the design of novel effective therapies for this deadly cancer. TRIM11 expression is upregulated, and it functions as an oncogenic protein in malignant gliomas [13] and lung cancer [14]. In this report, we demonstrate for the first time that TRIM11 is a key player in CC progression; we also show that downregulation of miR-24-3p is at least partly responsible for the upregulation of TRIM11 in CC cells. This novel miR-24-3p/TRIM11 axis may be useful for the development of new strategies for treating patients with CC.

We demonstrated that the mRNA and protein levels of TRIM11 were significantly increased in CC by performing data mining, qPCR analysis, and western blotting. High *TRM11* expression was associated with poor outcomes in patients with CC, indicating that the high level of TRIM11 was likely to present prognostic value. To explore the mechanism underlying the increased expression of TRIM11 in CC cells, we showed that miR-24-3p was at least one of the negative regulators of TRIM11. Gao Y et.al [18] showed that miR-24-3p was downregulated in human CC and was a prognostic factor for OS and DFS of CC patients. Transfection of miR-24-3p mimics significantly decreased cell number in SW480 and HT29 cells [18]. Our data also concurred with previous studies that miR-24-3p could inhibit cell proliferation in CC. Our data demonstrate that miR-24-3p directly targets TRIM11, suppressing cell proliferation, which suggests that miR-24-3p may play a suppressor role by targeting TRIM11.

E3 ubiquitin ligases are a large family of proteins that catalyze the ubiquitination of many proteins for targeted degradation by the 26S proteasome and play a key role in carcinogenesis [26]. E3 ubiquitin ligases are important regulators of a variety of biological processes including cell cycle regulation, cell proliferation, and apoptosis [27, 28]. The TRIM family of genes has been widely studied due to their key roles in development, differentiation and host cell antiviral defenses; however, roles in cancer biology are emerging [29]. For example, TRIM24 is an oncogenic transcriptional activator in prostate cancer [30]. TRIM15 expression is decreased in human colon adenocarcinoma compared with normal colon tissues; restoring expression in CC cells suppressed tumor growth in mice [31]. Our data shows that overexpression of TRIM11 promoted CC cell proliferation and inhibited apoptosis; in contrast, knockdown of TRIM11 using CRISPR/Cas9-mediated gene editing technology suppressed cell proliferation and induced apoptosis. Furthermore, knockdown of TRIM11 suppressed tumor growth *in vivo*. Those data indicated that TRIM11 functions an oncogene in CC.

In summary, our findings demonstrate that TRIM11 plays an oncogenic role and could serve as a clinical predictor in CC. We also highlight that TRIM11 expression dysregulation involves the miR-24-3p-mediated machinery, all of which offer potential avenues for the treatment of this fatal disease. Together, these data provide new insights into the molecular basis of this deadly malignancy.

## MATERIALS AND METHODS

### Cells and reagents

Nine human colorectal cancer cell lines (HCT116, HT29, SW480, SW620, DLD-1, LoVo, HCT8, RKO, and CaCo2) and the human colon fibroblast cell line CCD18-Co were purchased from the American Type Culture Collection (ATCC) and cultured according to their instructions. All cell lines used in this study were authenticated through short tandem repeat profiling less than 6 months ago when this project was initiated, and the cells have not been in culture for more than 2 months.

### Plasmids

The full-length cDNA of human TRIM11 was cloned into the pSin-puro vector (Focus Bioscience Co., Ltd, Nanchang, China). sgRNA-NC targeting EGFP (with no known targets in the human genome) was cloned intothe lentiCRISPR V1 vector; sgRNA-NC: GGGCGAGGAGCTGTTCACCG. TRIM11 single guide RNA (sgRNA) plasmid ligated into the lentiCRISPR V1 vector (Focus Bioscience Co., Ltd, Nanchang, China); sgRNA#1: 5’-ACGCATCCTGCATCTGCTTC-3’; sgRNA#2: 5’-TGCGTTGCTGTTCCAAGCCC-3’; sgRNA#3: 5’-CGGATGAGACCTGCG TCTTG-3’. The three sgRNAs targeting the exons of TRIM11 gene were selected from a published database of predicted high-specificity protospacer-adjacent motif target sites in the human exome [25]. The full-length 3′UTR of TRIM11 was cloned by standard procedures into the pGL3.0-control vector (Promega), immediately downstream of the stop codon of the luciferase gene to generate the TRIM11-Luciferase-WT luciferase reporter plasmid (Focus Bioscience Co., Ltd, Nanchang, China). Mutagenesis of the TRIM11-Luciferase-WT was performed using a QuikChange Site-Directed Mutagenesis kit (Stratagene, La Jolla, CA, USA).

### Antibodies

Anti-GAPDH was procured from Nanchang Focus Bioscience Co., Ltd. Anti-TRIM11 (HPA001209) was obtained from Sigma-Aldrich.

### Stable cell lines

pSin-puro-TRIM11, pSin-puro-empty vector, lentiCRISPR-NC, lentiCRISPR-sgRNA#1, lentiCRISPR-sgRNA#2 or lentiCRISPR-sgRNA#3 was co-transfected with pMD.2G and psPAX2 into HEK-293T cells for 48 h. The recombinant viruses were subsequently collected and added to CRC cells cultured with 8 μg/ml polybrene for 24 h. The stable lines were selected with 1μg/ml puromycin for two weeks (Focus Bioscience Co., Ltd, Nanchang, China).

### RNA extraction and qRT-PCR

These procedures were performed as previously described [32]. Briefly, total RNA was isolated using TRIzol reagent (Invitrogen) according to the manufacturer's instructions. First-strand cDNA was synthesized using Revert Aid™ First Strand cDNA Synthesis Kit (MBI Fermentas). The primers employed for amplifying TRIM11 and GAPDH were validated. TRIM11 primers are as follows: GAPDH primers are as follows:F 5’-ACAGTCAGCCGCATCTTCTT-3’ and R 5’-GACAAGCTTCCCGTTCTCAG-3’. TRIM11 primers are as follows:F 5’-GAGAACGTGAACAG GAAGGAG-3’ and R 5’-CCATCGGTGGCACTGTA GAA-3’. The expression of miR-24-3p was quantitated in human tissues using the mirVana qRT-PCR miRNA Detection Kit and the miR-24-3p and U6 snRNA primer sets (Ambion) in a Roche LightCycler (Roche, Basel, Switzerland).

### Cell proliferation and cell viability assays

*In vitro* cell proliferation was assessed using the CCK-8 assay; cells were seeded in 96-well plates at a density of 1,000 cells/well and incubated for 1, 2, 3, 4, or 5 days. Ten microliters of the CCK-8 reagent (Cell Counting Kit-8, Beyotime, China) was then added to each well, followed by incubation for 1.5 h. The absorbance value (OD) of each well was measured at 450 nm. For each experimental condition, 6 wells were used.

### Transient transfection for miRNAs mimics and inhibitors

The miRNA mimics (miR-24-3p, miR-101-3p, miR-148a, miR-15a, miR-504, miR-329, miR-370, miR-149, miR-320a, miR-193b, miR-544a, miR-150, miR-124-3p), miRNA control, and miRNA inhibitors for miR-24-3p were purchased from Ribobio (Ribobio, Guangzhou, China). After seeding into 6-well plates, cells were transfected with miRNA mimics at a final concentration of 100 nM using RNAiMAX Reagent (Invitrogen).

### Western blotting

These procedures were performed as described previously [32–34]. The gels were run under the same experimental conditions.

### Colony formation assay

Cells were plated in 6-well culture plates at 250 cells per well. Each group included 3 wells. After incubation for 15 days at 37°C, the cells were washed twice with PBS and stained with Giemsa solution (Focus Bioscience Co., Ltd, Nanchang, China). The number of colonies containing ≥50 cells was counted under a microscope.

### Flow cytometry

Apoptosis analysis was conducted with an Annexin V-FITC Apoptosis Detection Kit (KeyGen Biotech, China) according to the manufacturer's protocol. The percentage of apoptotic cells was determined using FACS flow cytometry and associated software (BECKMAN).

### Clinical data set analysis

Kaplan-Meier survival curves for TRIM11 were obtained using the tools at http://www.canevolve.org based on the GSE17536 data set, probe set 226566_at.

### Luciferase assay

This process was carried out as described previously [33, 35]. Briefly, the cells were plated in 12-well plates at the density of 2 × 10^5^ per well, and were transfected with 0.8 μg of luciferase plasmid. To normalize transfection efficiency, the cells were cotransfected with 8 ng of pRL-CMV (Renilla luciferase). After transfection for 48 hrs, luciferase activity was measured using the Dual-Luciferase Assay kit (Promega). Three independent experiments were performed, and their calculated means and standard deviations are presented.

### Study approval

The use of human CC tissues was reviewed and approved by the ethics committee of Jiangxi Cancer Hospital and was performedin accordance with approved guidelines. Informed consent was obtained.

### Statistical analysis

All statistical analyses were performed using SPSS for Windows, version 16.0(SPSS). Pearson's correlation analysis was performed to assess the relationships between miR-24-3p and TRIM11 in the tissues using mRNA expression data from qRT-PCR. All values from the *in vitro* assays are expressed as the mean±SD or SEM of at least three independent experiments or replicates. P values were calculated using the two-tailed Student's test. A p-value< 0.05 is considered statistically significant.

## SUPPLEMENTARY FIGURES


