# Supplementary material for: TRIM11, a direct target of miR-24-3p, promotes cell proliferation and inhibits apoptosis in colon cancer
Source: Oncotarget. 2016 Nov 24;7(52):86755–65. doi: 10.18632/oncotarget.13550 (PMC5349951; doi:10.18632/oncotarget.13550)
Supplement: Supplementary file 1 [file oncotarget-07-86755-s001.pdf]

TRIM11, a direct target of miR-24-3p, promotes cell proliferation and inhibits apoptosis in colon cancer

SUPPLEMENTARY FIGURE

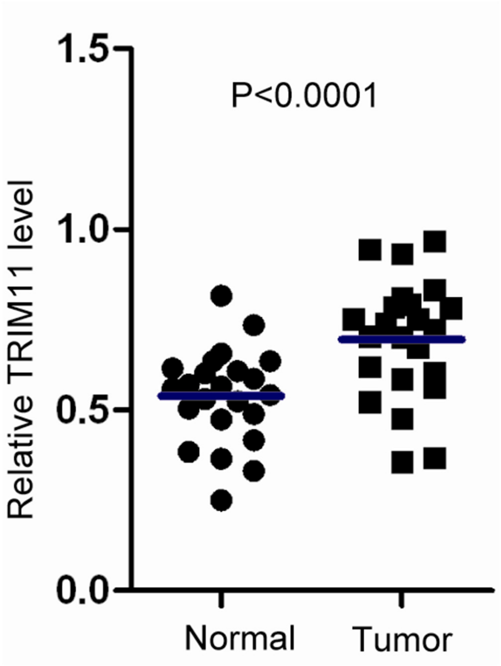

Supplementary Figure S1: Quantitative and statistical analysis of TRIM11 expression in clinical CC samples in tumor and paired normal tissues. The blue bars stand for the mean value. The p-value was calculated according to the raw data using Student's t-test ($P<0.0001$).
